# The Application of tDCS to Treat Pain and Psychocognitive Symptoms in Cancer Patients: A Scoping Review

**DOI:** 10.1155/2024/6344925

**Published:** 2024-04-13

**Authors:** Benedetta Capetti, Lorenzo Conti, Chiara Marzorati, Roberto Grasso, Roberta Ferrucci, Gabriella Pravettoni

**Affiliations:** ^1^Applied Research Division for Cognitive and Psychological Science, European Institute of Oncology IRCCS, Milan, Italy; ^2^Department of Oncology and Hemato-Oncology, University of Milan, Milan, Italy; ^3^I Neurology Clinic, ASST-Santi Paolo e Carlo University Hospital, Milan 20142, Italy

## Abstract

**Background:**

The use of transcranial direct current stimulation (tDCS) to modulate pain, psychological aspects, and cognitive functions has increased in recent years. The present scoping review aims to investigate the use of tDCS in cancer patients and its significant impact on psychocognitive and pain related symptoms.

**Methods:**

From the earliest available date to June 2023, a comprehensive search was conducted in three electronic scientific databases—PubMed, Scopus, and Embase—and other supplementary sources. Ten relevant studies were identified and included, comprising single case studies, randomized controlled trials, pilot studies, and one retrospective study. PRISMA guidelines for scoping reviews were followed.

**Results:**

These studies investigated the use of tDCS to improve pain and psychocognitive aspects in patients with various types of cancer, including breast, oral, bladder, lung, pancreatic, head and neck cancer, hepatocellular carcinoma, and meningioma. Overall, the results suggest that tDCS has shown efficacy in relieving pain, reducing anxiety and depression, and improving cognitive function in cancer patients.

**Conclusion:**

Due to the limited number and high heterogeneity of the existing literature in this field, more investigation and the establishment of standardized protocols would be required to obtain more conclusive evidence.

## 1. Introduction

The use of new technologies is assuming a prominent role within clinical practice, thus allowing a more targeted management of the patient by increasingly personalizing the experience within the clinical pathway. In particular, in the last decades, the use of noninvasive brain stimulation (NIBS) has seen a rapid growth in both research and clinical domains [1]. The most commonly used forms of NIBS include transcranial magnetic stimulation (TMS), which is based on the principles of electromagnetism, and transcranial electrical stimulation (tES), in which a low-level electrical current is applied to the scalp [2].

Both these NIBS can modify the brain activity through plastic reorganization processes [2–4]. Due to this reason, NIBS has been applied clinically to improve abnormal brain function in several psychiatric and neurological conditions [5]. The frequent therapeutic applications include the treatment of depression [6, 7], schizophrenia [8], posttraumatic stress disorder rehabilitation of aphasia or motor function after a stroke [9, 10], neurodegenerative diseases (e.g Alzheimer's and Parkinson's disease) [11, 12], obsessive–compulsive disorders [13], and chronic pain [14]. The operating principle of tES consists of a low-intensity electrical current between two electrodes placed on the surface of the scalp [15]. The procedure is often imperceptible to the subjects receiving tES and does not cause any clearly perceivable effects, except for local effects of stimulation (i.e. confined to the region under the electrodes) consisting of a slight tingling or heat sensation, which may rarely occur [3, 16].

This feature makes tES particularly suitable for use in studies that require sham conditions, a control condition where the subject is unaware that he is not receiving electrical stimulation [3].

The typology of tES most commonly used is the transcranial direct current stimulation (tDCS) [3, 16, 17]. It consists of a device battery delivering a low-intensity direct electrical current on the scalp through a pair of electrodes, an anode and a cathode [18, 19]. The shape of electrodes is generally round or rectangular with a diameter/diagonal ranging from 2 to 3.5 cm. To stimulate a precise cortical zone, the anode is placed over the selected zone, generally identified by an EEG headset (e.g., 10/20 System Positioning) [20]. tDCS is generally safe, but it can have potential side effects, including skin lesions [21], tingling, and mild pain [22]. Factors influencing the risk of side effects include stimulation intensity, skin impedance, and stimulation duration [23]. To ensure safety during tDCS stimulation, recommended safety parameters [24] include keeping the current below 2.5 mA [25], utilizing electrodes to minimize the risk of skin burns at the specified current intensity [26], limiting each session's current application duration to 20–60 min, and conducting sessions no more than twice daily [27].

Compared to the TMS, the tDCS has several advantages that have enabled it to be used in a large number of clinical and research studies. It is cheaper, has minimal side effects, is easier to apply and use, the device is portable and space saving, and has the possibility of home use that allows a primary role in telehealth programs [16].

It is also necessary to apply the right NIBS depending on the intervention, to enhance its clinical effectiveness [28]. The tDCS has profound effects on widespread functional connectivity, and the position of the electrode seems critical in mediating any effects on network connectivity and neuroplastic effects [3, 28]. Therefore, depending on the placement of electrodes, it is possible to act on clinical and psychocognitive symptoms caused by different medical disorders.

In recent years, improvements in cancer care through innovative and more effective treatments have contributed to a longer life expectancy for cancer patients, generating however the onset of long-term consequences. Cancer survivors, indeed, have to manage different clinical needs including psychocognitive alterations and pain management, that harm patients' quality of life [29, 30].

Given the efficacy of the application of NIBS on these symptoms in other typologies of patients, as listed above, the use of these noninvasive stimulation techniques in oncology may be relevant to the adoption of more specific therapies in a precision medicine context and e-health approaches [31, 32].

Despite the large number of studies using tDCS for the treatment of various medical disorders, its application in oncology is still limited.

Recently, however, a few studies have emerged investigating its application in certain typologies of cancer patients, focusing mainly on pain modulation [20, 32].

In particular, the use of tDCS can provide significant relief of different types of pain, including neuropathic and nociceptive pain [32–34].

Moreover, thanks to their advantages, the application of tDCS is also extending to the cancer palliative care. In this setting, patients present often mobility difficulties, and the adoption of tDCS allows to perform the stimulation at the patient's bedside or to promote the application of home delivery tDCS [20]. What is expected is that the use of these NIBS can lead to a decrease in the length of hospital stay and a significant reduction of analgesic drug consumption [20].

Additionally, the development of telemedicine through remote treatment techniques can have a significant economic impact, reducing healthcare costs [35, 36].

However, the specific mechanisms of action of the tDCS on the modulation of psychological distress, cognitive deficits, and pain related to cancer, are still uncertain [32].

In this scenario, the objective of the current scoping review is to investigate the application of tDCS for the management of pain, cognitive symptoms, and psychological aspects in oncology patients, based on the available literature.

## 2. Methods

The scoping review methodology has been selected to comprehensively delve into the literature and identify gaps in the application of tDCS in cancer patients. This approach is suitable for investigating broad research questions and gaining a thorough understanding of existing evidence without excluding studies based on their methodological quality [37].

The current scoping review followed the guidelines outlined in the preferred reporting items for systematic reviews and meta-analysis extension for scoping review (PRISMA-ScR) [38].

### 2.1. Eligibility Criteria

The eligibility criteria for studies in this review were determined based on the evaluation of tDCS use in cancer patients, inclusion of adult participants (aged >18 years), original research articles, and publication in English. Conversely, studies were excluded if they involved a different NIBS technique, lacked reported results, or were review, meta-analyses, discussion papers, editorials, or conference abstracts.

### 2.2. Information Sources and Search Strategy

A comprehensive literature search was conducted, including articles from the early stages through June 2023, using the following electronic databases: PubMed, EMBASE, and Scopus. For supplementary references, we also searched the reference lists from the selected papers. In the research, unpublished sources, including conference abstracts, clinical trials, and ongoing controlled studies, were also included. The developed search strategy integrated MeSH terms and keywords, covering “tDCS,” “cancer,” “pain,” and “psychocognitive aspects.” These terms were subsequently refined for synonyms, and the resultant search string was employed across all three databases under consideration. A detailed description of the search strategy applied across all databases is provided in Supplementary 1.

### 2.3. Selection of Sources of Evidence

After all, duplicates had been removed, two independent authors (BC and LC) reviewed the title and abstract of all potentially relevant studies, classifying them as “included,” “excluded,” or “maybe,” following the preestablished admission criteria. Subsequently, the abstracts classified as “included” and “maybe” were compared by the reviewers to determine which articles should undergo full-text review. For each selected abstract, the full article was retrieved and independently assessed by the authors (BC and LC). In the event of disagreement between the authors at both stages, a third author (CM) was consulted to reach a mutual agreement.

### 2.4. Data Charting Process

Data retrieval from studies meeting the inclusion criteria were independently conducted by two authors (BC and LC). Information from the integrated studies, including author(s), publication year, study location, participant demographics (such as age and cancer type), the research design, methods employed for tDCS, and the main results, were extracted using Microsoft Excel 2016.

## 3. Results

### 3.1. Study Selection

**Figure 1:**
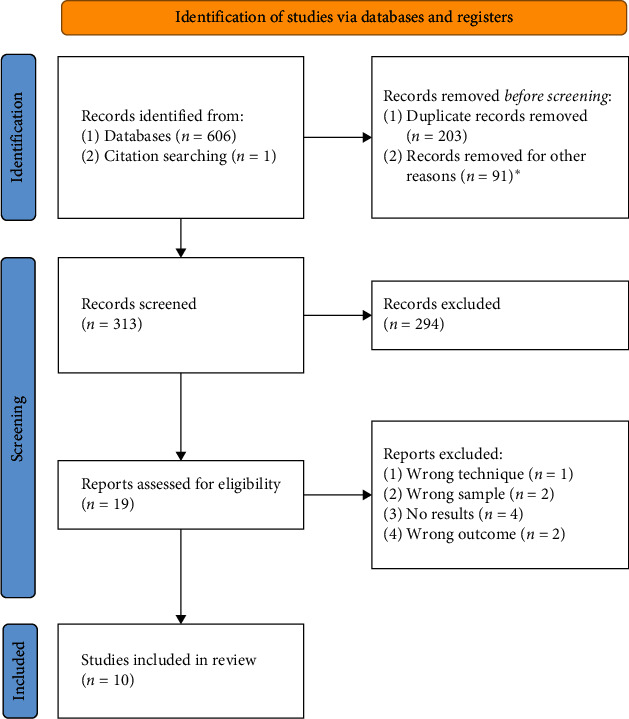
PRISMA flow diagram for the review process.  ^*∗*^Record removed for other reasons: review, meta-analyses, discussion papers, editorials, or conference abstracts.

As illustrated in the PRISMA flow diagram of the study selection (Figure 1), a total of 606 articles were initially identified from the databases, and an additional publication from alternative sources was considered potentially eligible. Following the removal of duplicates, reviews, and meta-analyses, 313 articles underwent screening based on title and abstract. Out of these, 294 studies were excluded. A comprehensive full-text analysis was then conducted on 14 articles, three letters to the editor, and two abstracts by BC. Subsequently, after completing the full-text analysis, nine studies were excluded from the scoping review as they did not meet the eligibility criteria. The entire process underwent a review by a second author (LC) to confirm the eligibility of the selected studies.

### 3.2. Overview of the Studies

After checking for duplications and ensuring compliance with the selection criteria, 10 studies were included, comprising seven original research studies and three letters to the editor. All included studies were conducted in adults. The oldest publication dated back to 2007, whereas the latest were published in 2022.

The predominant studies encompassed individual case studies (*n* = 4) [39–42], randomized controlled trials (*n* = 3) [43, 44], and pilot studies (*n* = 2) [34, 45]. Additionally, a retrospective study was included [32]. Most studies were conducted in the United States (*n* = 3) [34, 42, 45] followed by Egypt (*n* = 2) [43, 44], and one each from China [42], France [39], Serbia [46], Poland [40], and Brazil [41].

The studies greatly varied in the sample size, ranging from 1 to 98 participants. Particularly, in four studies, there was only one participant [39–42], while five studies had a sample size greater than or equal to 40 participants [34, 43, 44, 46, 47]. Finally, one study had 16 participants [45].

Three studies looked at populations of women with breast cancer [42, 43, 45]. The remaining studies looked at homogeneous groups of cancers including patients with oral cavity cancer [47], bladder cancer [39], head and neck cancer [34], hepatocellular carcinoma [44], lung cancer [46], another on patients with meningioma [40], and finally a last one on pancreatic cancer [40]. In addition, tDCS stimulation was used alone in all included studies, except for one study [40] in which tDCS was used in combination with neurofeedback. tDCS was mostly used in daily–weekly sessions, ranging from 5 to 10 consecutive days. Only one study [43] provided a single session of tDCS. Only one study [45] investigated the presence of potential side effects following tDCS treatment.

The characteristics of the studies are summarized in Table 1.

The target cortical areas varied among studies. The main target areas were the primary motor cortex [34, 41, 43, 44, 46] and the prefrontal cortex [39, 40, 42, 45]. Regarding the tDCS protocol, the duration of stimulation ranged between 20 [34, 39, 42, 43, 46] and 30 min [44, 47], with only one study using 15 min [45]. The intensity of stimulation was 2 mA in five studies [34, 42–44, 47], 1 mA in two studies [39, 45], and, respectively, 1.2 mA [47] and 10 mA [41] in the last studies.  Table 2 summarizes the tDCS parameters of the included studies. The list of tDCS devices and their specifications used in the reviewed papers is summarized in Supplementary 2.

We did not perform a quality appraisal of the included studies due to the significant heterogeneity in study designs, which would have made it challenging to directly compare their findings. Our objective was to comprehensively summarize the extent and full range of evidence on the topic.

### 3.3. Outcomes and Measures

The psychological domain was the most frequently studied outcome, with seven articles [39, 40, 43–47] out of 10 total. Specifically, the domain mainly studied was depression [39, 40, 43, 45–47], followed by anxiety [39, 41, 46, 47], sense of well-being, and sense of malaise [43].

Instead, pain has been evaluated in a total of six studies [34, 39, 41, 43, 44, 46]. Finally, three studies [41, 42, 45] evaluated cognitive functions such as attention [41, 42, 45] memory [41, 42], and executive functions [42]. Self-report measure of difficulty with memory, attention, concentration, language, and thinking abilities was also evaluated [45].

In Supplementary 3, a summary of the questionnaires employed to assess each domain is provided.

### 3.4. tDCS Effects on Psychocognitive and Clinical Outcomes

The explored outcomes included the use of tDCS for pain management, psychological aspects, and cognitive functions in oncology patients. Specifically, studies have reported a reduction in perceived pain following the use of tDCS [34, 39, 41, 44, 46]. Only one study found no improvement in pain following a single session of tDCS [43].

Within the chosen studies, the researchers examined the changes in depression and anxiety levels before and after tDCS sessions, and in most cases [39, 40, 44, 47], the results reported decreased levels of anxiety and depression following stimulation. Only in two cases [45, 46], there were no changes in these variables after the use of tDCS. Improved sense of well-being and reduced patient-perceived discomfort after stimulation with tDCS also emerged [43]. Finally, some studies have reported an overall improvement in cognitive function [42, 45] and a subtle improvement in subjective experience of cognitive function [45] following tDCS stimulation, with the exception of one study [41]. Specifically, the results of one study [39] demonstrated greater sustained attention in the tDCS group compared to sham stimulation in patients with cancer-related self-reported cognitive dysfunction. Contrary to this result, another study showed improvement in memory and executive functions, but not in attention following stimulation with tDCS [42].

Table 3 presents a summary of the prevailing outcomes derived from the selected studies.

## 4. Discussion

The objective of this review was to explore the application of tDCS for the management of pain and psychocognitive aspects in patients diagnosed with various types of cancer. The literature on this topic is still limited. However, in most cases, the analyzed studies have highlighted that tDCS can lead to significant improvements in pain, anxiety, depression, and cognitive functions in cancer patients. This is a relevant finding because these symptoms are often seen in cancer patients and can adversely affect their overall quality of life [48–50].

Several positive outcomes have been observed, providing evidence of the potential beneficial impact of tDCS in these areas. However, it is important to note that the availability of data is more focused on the use of tDCS for psychological aspects and pain, while evidence regarding cognitive aspects in cancer patients is more limited. Specifically, pain reduction has been reported in case studies [39, 45], two randomized studies [44, 46], and a pilot study [34]. Of particular, significance is that one study reported conflicting results [43] but, overall, the positive effects of tDCS on pain reduction have been promising and this can contribute to better symptom management and overall well-being of patients [51]. In particular, the study by Ibrahim et al. [44] showed that pain began to decrease more consistently after the tenth tDCS session, suggesting that intensified protocols are more effective. This finding is consistent with other studies that have shown repeated tDCS sessions to be more effective in reducing pain, leading to more long-lasting outcomes [52, 53].

Additionally, the review has highlighted that tDCS can positively influence psychological aspects, such as depression and anxiety, in cancer patients. Most of the studies have demonstrated a reduction in levels of depressive and anxious symptoms after tDCS treatment [39, 40, 44, 47], still a case study [41] and a randomized study [46] did not report significant changes in these aspects. An important consideration is that the initial level of depression and anxiety in participants may vary across studies, and this variability, combined with the utilization of different stimulation methodologies, can lead to differing outcomes after tDCS [54]. Nevertheless, in general, the positive effect of tDCS on mental health may contribute to greater psychological resilience during the care journey of cancer patients [55].

Furthermore, the potential for improving cognitive functions such as attention, memory, and executive functions through the use of tDCS in cancer patients has emerged [42, 45]. However, the results are not without exceptions. A single case study [41] has reported different results, emphasizing the need for further research and exploration of individual differences in the response to tDCS sessions [56].

Overall, this scoping review reported results consistent with other studies in the literature involving different patient populations. For example, studies conducted on patients with fibromyalgia highlighted how tDCS can be considered a safe and effective therapeutic option for the treatment of pain [57] and depressive symptoms [58, 59]. Another study has shown similar results in patients with multiple sclerosis, reporting a reduction in pain following 5 days of tDCS treatment and demonstrating that this effect persisted beyond the stimulation period, leading to long-lasting clinical effects [60]. Finally, a study by Boggio et al. [61] highlighted how, following anodal tDCS session in patients with Parkinson's disease, a significant improvement in working memory was observed.

It is noteworthy that, in addition to implementing tDCS sessions, the literature [62–64] has demonstrated the effectiveness of other NIBS techniques, such as TMS, in managing pain and psychocognitive symptoms in cancer patients. In particular, it has been observed that repetitive transcranial magnetic stimulation (rTMS) significantly reduces the intensity of pain and depressive symptoms in patients with nonbrain malignancy tumors [65]. rTMS indeed has a greater and more focused electric field compared to tDCS [66]. On the other hand, some NIBS techniques, including cranial electrical stimulation (CES), do not show a significant reduction in pain and depressive symptoms in cancer patients [65]. However, tDCS remains the more widely adopted technique due to its advantages such as simplicity and potential for remote application, thus enabling more frequent sessions [16]. This aspect is important because, as mentioned earlier, it has been demonstrated that NIBS requires repetition to promote the long-term sustainability of the clinical outcome [67, 68]. Regular tDCS sessions may further induce lasting performance enhancements by fostering neuroplasticity and strengthening neural connections over extended periods [69].

## 5. Research and Practice Implications

Although the results presented are encouraging, further studies on the application of tDCS in cancer patients are needed to corroborate and generalize the results. In future endeavors pertaining to tDCS, spanning from research to clinical implementation, it is crucial to account for individual variances that may influence the impact of stimulation on pain perception and psychocognitive aspects. By doing so, we can effectively identify the specific conditions in which tDCS exhibits the highest efficacy in enhancing patients' performance and in reducing clinical symptoms. Indeed, various factors, such as age, personal characteristics, and education level, have been shown to influence the effects of tDCS [70–72]. Furthermore, numerous brain-related anatomical factors can also influence responsiveness to tDCS, and these factors may evolve as the brain develops [73]. Therefore, gaining a comprehensive understanding of the intricate relationships between these factors and improvements in pain and psychocognitive aspects will enable to maximize therapeutic benefits for patients with cancer.

In addition, remote administration of tDCS has demonstrated feasibility and effectiveness in improving pain management and psychocognitive aspects in other patient populations [74, 75]. Thus, protocols could be developed including the use of remote tDCS for pain and psychocognitive management in cancer patients, as well. Delivering stimulation at home would not only allow for repeated daily sessions that can lead to long-term performance benefits [67], but it would also circumvent the challenges associated with multiple hospital visits. The burden of frequent hospital visits poses significant barriers, including travel difficulties and access to care, with economic and logistical implications [76].

Furthermore, some studies [77, 78] have shown how tDCS, when combined with other treatments, such as cognitive training or physical exercise, can enhance their effects on pain and psychocognitive aspects. The introduction of a home-based approach that includes the use of remote tDCS and home rehabilitation could improve patient engagement in routine clinical care and reduce disparities in accessing healthcare services [79], thereby enhancing the physical and mental health of cancer patients. There is a need for renewed initiatives in tailoring telemedicine services to enhance patient empowerment, thereby optimizing both self-management and clinical outcomes [80]. This suggests the potential for opening new perspectives in the integrated treatment of cancer, offering holistic approaches that encompass both physical and psychocognitive dimensions of patient care. Indeed, tDCS, combined with other rehabilitation treatments, can be an effective aid in this sense, but each patient must be individually evaluated to find the most suitable treatment for their needs. Studying the effects of tDCS on different types of cancer could provide valuable information on the possible applications and benefits of tDCS in specific cancer conditions.

In summary, the use of tDCS in cancer patients has potential benefits on pain and psychocognitive that can be purposefully integrated into existing treatment protocols, with a focus on personalized treatment plans to maximize clinical outcomes and improve the quality of life of cancer patients. For instance, incorporating the use of tDCS remotely, in palliative care patients, could reduce the use of analgesic pain medications and decrease hospitalization time [20]. Customizing therapeutic approaches based on individual patient characteristics and responses to tDCS can optimize its effectiveness and contribute to reducing pain and psychocognitive symptoms in cancer patients.

However, despite its practical advantages and increasing utilization, tDCS faces methodological and conceptual challenges that may impede its widespread adoption. Key limitations include its low spatial and temporal resolution, poorly understood stimulation parameters, and variability in observed effects [81]. In addition, some current tDCS devices may be impractical for daily use by patients. Addressing these challenges will require advancements in electrode design, understanding of stimulation parameters, and rigorous investigation into the factors influencing tDCS outcomes to enhance its reliability and reproducibility. Moreover, tDCS devices could benefit from integrated real-time monitoring and feedback systems to evaluate the effectiveness of stimulation and make any corrections during treatment. This could allow more accurate tailoring of stimulation to the patient's individual response.

## 6. Conclusion

This review has highlighted how tDCS sessions have demonstrated their effectiveness in enhancing several critical aspects of cancer patient management. Specifically, it has been revealed that such sessions can lead to significant improvements in pain management, anxiety, and depression reduction, as well as enhancement of cognitive functions in cancer patients. These findings suggest a promising role for tDCS as an integral part of oncological treatment, contributing to overall well-being and improving the quality of life for patients facing complex challenges throughout their cancer care journey. Nonetheless, there is a demand for additional well-conducted research to set robust benchmarks, given the wide-ranging disparities in the chosen publications concerning methodology and sample composition, which hinder generalizing the findings and assessing the effectiveness of tDCS in cancer patients. This will enhance our comprehension of the mechanisms involved, identify the best stimulation protocols, and evaluate the long-term effectiveness of tDCS in the context of oncology.

## Figures and Tables

**Table 1:** Characteristics of included studies.

First author, year	Country	Sample size	Age range and/or mean (SD)	Cancer type	Study design
Gao et al., 2022 [47]	China	*N* = 72 tDCS group, *n* = 36 (29 M, 7 F) CG, *n* = 36 (31 M, 5 F)	tDCS group: 52.5 ± 8.6 CG: 53.1 ± 8.2	Oral cancer	RS

Gaynor et al., 2020 [45]	USA	*N* = 16 (F)	40–65 years	Breast cancer	PS

Knotkova et al., 2014 [42]	USA	*N* = 1 (F)	55 years	Breast cancer	SC

Nguyen et al., 2016 [39]	France	*N* = 1 (M)	80 years	Bladder cancer	SC

Stamenkovic et al., 2020 [46]	Serbia	*N* = 55 tDCS group, *n* = 27; (16 M, 11 F) Sham group, *n* = 28; (23 M, 5 F)	tDCS group: 61.44 ± 7.98 Sham group: 61.89 ± 5.79	Lung cancer	RCT

Hu et al., 2016 [34]	USA	*N* = 98 tDCS group, *n* = 5 (4 M, 1 F) CG, *n* = 93 (retrospective data)	62.6 ± 5	Head and neck cancer	PS

Kamal et al., 2022 [43]	Egypt	*N* = 60 (F) tDCS group, *n* = 30 Sham group, *n* = 30	tDCS group: 48.4 ± 6.6 Sham group: 50.4 ± 5.7	Breast cancer	RCT

Ibrahim et al., 2018 [44]	Egypt	*N* = 40 tDCS group, *n* = 20 (n.a.) Sham group, *n* = 20 (n.a.)	tDCS group: 58.9 ± 5.6 Sham group: 56.85 ± 9.16	HCC	RCT

Mirski et al., 2015 [40]	Poland	*N* = 1 (F)	45 years	Meningioma	SC

Silva et al., 2007 [41]	Brazil	*N* = 1 (F)	65 years	Pancreatic cancer	SC

*Notes*. tDCS = transcranial direct current stimulation; SD = standard deviation; USA = United States of America; CG = control group; *M* = male; *F* = female; n.a. = not applicable; HCC = hepatocellular carcinoma; RS = retrospective study; PS = pilot study; SC = single case study; and RCT = randomized controlled trial.

**Table 2:** tDCS parameters of included studies.

First author, year	Brain region	Stimulation intensity (mA)	Anodal electrode position	Cathodal electrode position	Duration (min)	Session numbers	Electrode sizes
Gao et al., 2022 [47]	—	2	—	—	30	28	—
Gaynor et al., 2020 [45]	Left dorsolateral prefrontal cortex	1	F3	F4	15	2	1 cm radius
Knotkova et al., 2014 [42]	Prefrontal cortex	2	F3	Contralateral supraorbital region	20	5	4.5 × 6 cm
Nguyen et al., 2016 [39]	Right prefrontal cortex	1	C3	F2	20	5	—
Stamenkovic et al., 2020 [46]	Left primary motor cortex	1.2	C3	Contralateral supraorbital region	20	5	1 cm radius
Hu et al., 2016 [34]	Primary motor cortex	2	C5	F4	20	20	25 cm^2^
Kamal et al., 2022 [43]	Primary motor cortex	2	C4	Opposite supraorbital region	20	1	35 cm^2^
Ibrahim et al., 2018 [44]	Primary motor cortex	2	C4	Opposite supraorbital region	30	10	35 cm^2^
Mirski et al., 2015 [40]	Left frontal cortex	—	F7	—	—	20	—
Silva et al., 2007 [41]	Primary motor cortex	10	C3	Contralateral supraorbital area	—	—	35 cm^2^

**Table 3:** Results of included studies.

First author, year	Procedure	Outcomes	Measures	Main results
Gao et al., 2022 [47]	tDCS for 30 min, once daily for 4 weeks	Anxiety; depression	SAS; SDS	Anxiety and depression: After treatment, tDCS group achieved a reduction in anxiety and depression

Gaynor et al., 2020 [45]	Four study visits over 4 consecutive days (2 days of tDCS stimulation and 2 days of sham stimulation)	Attention; self-report measure of difficulty with memory, attention, concentration, language, and thinking abilities; measure of difficulties with attention and concentration related to difficulty filtering irrelevant sensory information; patients' experience with tDCS; adverse events related to tDCS	CPT; PAOFI; SGI; tDCS patient experience questionnaire; brunoni adverse events questionnaire	Self-reported cognitive difficulties: A nominal decrease in self-reported cognitive problems: mean PAOFI score pre-tDCS (97.71 ± 25.54) vs. after tDCS (93.93 ± 21.90) sessions Marginally significant change in SGI scores from pre-tDCS (*M* = 62.14 ± 30.49) vs. after tDCS (*M* = 56.43 ± 29.22) Attention: Better sustained attention in tDCS group vs. sham, *p* < 0.05

Knotkova et al., 2014 [42]	T0: five sessions of tDCS on 5 consecutive days T1: 2 weeks after tDCS completion	Executive functioning; memory (nonverbal); attention; global cognitive score	Go/No-Go test, stroop interference test and catch game; immediate recognition and delayed recognition tests; score across the three cognitive domains	Improvements in all cognitive functions except attention. Global cognitive score: pre-tDCS: 88.7 after tDCS: 108.6 after 2 weeks: 103.9 Memory: pre-tDCS: 79.4 after tDCS: 114.9 after 2 weeks: 111.9 Executive functions: pre-tDCS: 89.5 after tDCS: 108.2 after 2 weeks: 101.3 Attention: pre-tDCS: 97.2 post-tDCS: 102.8 post 2 weeks: 98.5

Nguyen et al., 2016 [39]	T0: One tDCS stimulation (20 min) for 5 consecutive days	Pain; depression and anxiety; analgesic and coanalgesic consumption in chronic pain	VAS; HAD; MQS	Pain: Prestimulation: VAS score varying between 6 and 8/10 with at least four very painful peaks per day poststimulation: by the second day of treatment, VAS fluctuating between 2 and 3/10 with only 1–2 painful peaks per day Drug treatment: Pre-stimulation: 36 MQS score Post-stimulation: 15 MQS score Depression Prestimulation: 14/21 HAD score poststimulation: 6/21 HAD score
Stamenkovic et al., 2020 [46]	tDCS group received stimulation for 20 min on 5 consecutive days after thoracotomy vs. sham group T1: immediately before the thoracotomy T2: immediately after the thoracotomy T3–T6: every 1 hr for 4 hr Т7–Т31: every 6 hr for 5 days	Pain; depression; anxiety; morphine dose	VAS; BDI; PRO survey (questions on pain intensity, time with severe pain; pain interference, patient satisfaction)	Morphine dose: Cumulative morphine dose administered during the first 120 hr after surgery was lower by 31.25% (Cohen's *d* = 0.42) in the tDCS group (*p*=0.043) Pain: On postoperative day 5, VAS pain score with cough was significantly lower in the tDCS group (*p*=0.018) Pain intensity, time with severe pain, pain interference: Pain interference with cough (PROs) was 80% lower (*p*=0.013), and not identify any other differences existed between groups with regard to PRO Anxiety, depression, mood: There was no significant difference between group

Hu et al., 2016 [34]	Prestudy visit: EEG + full questionnaire packet 1 week of CRT: full questionnaire packet 2 week of CRT: five tDCS stimulation daily + EEG + questionnaires 3 week of CRT: five tDCS stimulation daily + EEG + questionnaires 4 weeks of CRT: three tDCS stimulation daily + questionnaires 5 weeks of CRT: three tDCS stimulation daily + questionnaires 6 week of CRT: two tDCS stimulation daily + questionnaires 7 weeks of CRT: two tDCS stimulation daily + EEG + full questionnaire Packet 1 week follow-up: EEG + full questionnaires packet 1 month follow-up: EEG + full questionnaires packet	Pain; weight loss; and graded dysphagia between the tDCS stimulus cohort and control cohort	VAS; PANAS	Pain: After tDCS: VAS reduced in every week (average decrease range: 0.19–0.57) and PANAS scores decreased (positive decrease range: −0.25–6.5, negative decrease range: 0.5–3.5)

Kamal et al., 2022 [43]	Before chemotherapy T0: measurements before tDCS one session of tDCS stimulation (20 min) or sham treatment After chemotherapy T1, T2, T3: measurements every 24 hr for 72 hr after cessation of chemotherapy	Patients' nausea; pain, malaise, and sense of well-being	Cumulative index of nausea, VAS-N, episodes of vomiting; ESAS	Pa**i**n: No significant difference in tDCS group vs. sham group. Malaise: tDCS showed a reduction in ESAS malaise score (*p* < 0.001) Sense of well-being: tDCS group improves sense of well-being score (*p* < 0.001) over 3 days vs. the sham group

Ibrahim et al., 2018 [44]	tDCS for 10 consecutive days (30 min) T0: baseline T1: after first tDCS T2: after fifth tDCS T3: after 10th tDCS T4: after 1 month from last tDCS.	Pain; depression	VDS; VAS; HAM-D	Pain: Pain reduction in tDCS group after T3 (VAS, *p*=0.001; VDS, *p*=0.008) and T4 (VAS, *p*=0.037, VDS, *p*=0.001). Depression: Depression reduction in tDCS group after T3 (*p*=0.001) and T4 (*p*=0.002)

Mirski et al., 2015 [40]	20 sessions of individually tailored anodal tDCs + neurofeedback twice a week	Depression	BDI	Depression: Before surgery: no depression (BDI score: 10) 1 month after surgery: mild depression (BDI score: 20) 6 months after surgery: severe depression (BDI score: 32) tDCS stimulation on depressive symptoms: After tDCS + neurofeedback: no depression (BDI score: 6) After 6 months of follow-up: no depression (BDI score: 7)

Silva et al., 2007 [41]	Sham and active tDCS in a randomized order	Pain; mood; anxiety; memory; attention; and cognitive functions	Numeric scales for pain, mood, and anxiety; MMSE, stroop test, forward and backward digit span; questionnaire for adverse effects	Pain, mood, anxiety: Sham group: no changes tDCS group: pain reduction from 4 to 0; no changes in mood and anxiety after tDCS. Cognitive effects: Sham group: MMSE and digit span forward: no changes Digit span backward: increase from 2 to 3 (row scores) Stroop colors performance execution time decreased tDCS group: MMSE and digit span forward and backward: no changes Stroop colors execution time decreased from 23.06 to 20.56 s (row scores)

*Notes*. tDCS = transcranial direct current stimulation; SAS = self-rating anxiety scale; SDS = self-rating depression scale; CPT = Conners' continuous performance test; PAOFI = patient assessment of own functioning inventory; SGI = sensory gating inventory; VAS = visual analogue scale; HAD = Hamilton anxiety and depression scale; MQS = medication quantification scale; BDI = beck depression inventor; VAS-N = visual analog scale for nausea; ESAS = Edmonton symptoms assessment scale; VDS = verbal descriptor scale; HAM-D = Hamilton rating scale for depression; MMSE = mini-mental state examination; CRT = chemoradiotherapy; PANAS = McGill and positive and negative affect schedule.

## Data Availability

Data sharing is not applicable to this article as no datasets were generated or analyzed during the current study. The search string is detailed in the supplemental material.

## Additional Points

*Limitations*. This study is subject to several limitations; thus, it is important to interpret our results with caution. In particular, the inclusion of heterogeneous samples of oncological patients encompassing various types of cancer does not allow for an accurate comparison of results. Additionally, although we employed a standardized procedure for the revision process, our selection criteria included only English-language articles. Therefore, we cannot exclude the possibility that other studies may have yielded different results. Moreover, there are several limitations of the studies included in this review. First, the small sample sizes in these studies render them nonrepresentative, potentially limiting the generalizability of their findings, and consequently impacting the reliability of the conclusions drawn. Furthermore, the absence of follow-up assessments to evaluate the long-term effects of tDCS is notable. Such assessments could offer valuable insights into the sustained efficacy and safety of the intervention over time, as well as potential risks associated with prolonged or repeated tDCS sessions. Indeed, without such follow-up assessments, our understanding of the clinical utility and risk profile of tDCS interventions could remain incomplete, underscoring the need for more comprehensive research in this area. Second, despite a greater emphasis on psychological aspects and pain, cognitive aspects received limited attention in these studies. This is noteworthy, given the existing literature reporting cognitive impairments in cancer patients both before and after chemotherapy treatment [82–85]. In studies exploring cognitive aspects, despite the presence of explicit guidelines [48] regarding the assessment of cognitive functions, the collective studies employed varied tools, and the potential learning effect from repeated administration of cognitive tests is not addressed. This represents a limitation, as the improvement of cognitive functions may have been influenced by this aspect. Lastly, another potential limitation of the included studies is the fact that almost none of them have investigated and reported the presence or absence of potential side effects associated with tDCS treatment in cancer patients. Therefore, it is necessary for future research to actively investigate the presence or absence of side effects, including through the creation of specific questionnaires.

## Supplementary Materials

Supplementary 1 Search strategy for scientific literature.

Supplementary 2 tDCS devices and their specifications.

Supplementary 3 Questionnaires used to assess psycho-cognitive and clinical domains.
