# Supplementary material for: The Application of tDCS to Treat Pain and Psychocognitive Symptoms in Cancer Patients: A Scoping Review
Source: Neural Plast. 2024 Apr 13;2024:6344925. doi: 10.1155/2024/6344925 (PMC11032211; doi:10.1155/2024/6344925)
Supplement: Supplementary 1 — Search strategy for scientific literature. [file 6344925.f1.docx]

***Supplementary material 1*** *Search Strategy*

| **SEARCH STRATEGY FOR SCIENTIFIC LITERATURE** | | | | |
| --- | --- | --- | --- | --- |
| **Search Engine:** | **Search String:** | **Hits** | **Relevant^a^** | **Included^b^** |
| Pubmed | (("Transcranial Direct Current Stimulation"[Mesh] OR "transcranial direct current" OR "transcranial electrical" OR "transcranial random noise" OR "tdcs" OR "tdcss") AND (neoplasms OR cancer OR tumor OR tumour OR oncological OR malignancy OR carcinoma)) AND (pain OR cognitive OR cognition OR psychology OR psychological OR anxiety OR depression) | 87 | 13 | 9 |
| Embase | ('neoplasm'/exp OR 'neoplasm' OR 'cancer'/exp OR 'cancer' OR 'tumor'/exp OR 'tumor' OR 'tumour'/exp OR 'tumour' OR 'oncological' OR 'malignancy'/exp OR 'malignancy' OR 'carcinoma' OR 'carcinoma'/exp OR carcinoma) AND ('transcranial direct current stimulation'/exp OR 'transcranial direct current stimulation' OR 'transcranial direct current' OR tdcs OR tdcss OR 'transcranial electrical'/exp OR 'transcranial electrical' OR 'transcranial random noise'/exp OR 'transcranial random noise') AND ('pain' OR 'pain'/exp OR pain OR 'cognition' OR 'cognition'/exp OR cognition OR cognitive OR 'psychology' OR 'psychology'/exp OR psychology OR psychological OR 'anxiety' OR 'anxiety'/exp OR anxiety OR 'depression' OR 'depression'/exp OR depression) | 296 | 16 | 9 |
| Scopus | ( ( ( "transcranial direct current stimulation" OR "transcranial direct current" OR "transcranial electrical" OR “transcranial random noise” OR "tdcs" OR "tdcss" ) AND ( neoplasms OR cancer OR tumor OR tumour OR oncological OR malignancy OR carcinoma ) ) AND ( pain OR cognitive OR cognition OR psychology OR psychological OR anxiety OR depression ) ) | 223 | 15 | 9 |
| From other sources |  | 1 | 1 | 1 |
| **Subtotal** |  | 607 | 45 | 28 |
| **Duplicates** |  |  |  | 18 |
|  |  |  |  |  |
| **Total** |  | 607 | 45 | 10 |
| ^a^ Relevant: number of relevant articles based on title, abstract, and keywords | | | | |
| ^b^ Included: number of included articles based on full article | | | | |
