# Supplementary material for: The Application of tDCS to Treat Pain and Psychocognitive Symptoms in Cancer Patients: A Scoping Review
Source: Neural Plast. 2024 Apr 13;2024:6344925. doi: 10.1155/2024/6344925 (PMC11032211; doi:10.1155/2024/6344925)
Supplement: Supplementary 2 — tDCS devices and their specifications. [file 6344925.f2.docx]

**Supplementary material 2** *tDCS devices and their specifications.*

| **First Author, year** | **tDCS Device** | **Technical Specifications** |
| --- | --- | --- |
| Gao et al., 2022 | Transcranial Ltd., London, United Kingdom | battery-powered microprocessor-controlled constant current device |
| Gaynor et al., 2020 | Neuroelectrics, Barcelona, Spain | hybrid EEG/tES multichannel transcranial current stimulator |
| Knotkova et al., 2014 | - | - |
| Nguyen et al., 2016 | Starstim, Barcelona, Spain | - |
| Stamenkovic et al., 2020 | Neuroelectrics, Barcelona, Spain | wireless Starstim tDCS neurostimulator |
| Hu et al., 2016 | Neuroelectrics, Barcelona, Spain | - |
| Kamal et al., 2022 | NeuroConn Gmbh, 98693 llmenau, Germany | battery-driven DC stimulator |
| Ibrahim et al., 2018 | NeuroConn Gmbh, 98693 llmenau, Germany | battery-driven DC stimulator |
| Mirski et al., 2015 | - | - |
| Silva et al., 2007 | - | - |

*Note: EEG=electroencephalogram; tES= transcranial electrical stimulation.*
