# Supplementary material for: The Application of tDCS to Treat Pain and Psychocognitive Symptoms in Cancer Patients: A Scoping Review
Source: Neural Plast. 2024 Apr 13;2024:6344925. doi: 10.1155/2024/6344925 (PMC11032211; doi:10.1155/2024/6344925)
Supplement: Supplementary 3 — Questionnaires used to assess psycho-cognitive and clinical domains. [file 6344925.f3.docx]

**Supplementary material 3** *Questionnaires used to assess psycho-cognitive and clinical domains.*

| **Domain** | **Questionnaires** |
| --- | --- |
| *Depression* | - BDI - SDS - HAM-D |
| *Anxiety* | - SAS - HADS - VAS |
| *Well-being* | - ESAS |
| *Pain* | - VAS - ESAS - PANAS |
| *Cognitive functions* | - MMSE - CPT - Go/No-Go test - Stroop - Immediate Recognition and Delayed Recognition - Catch Game - PAOFI |

*Note: BDI= Beck Depression Inventory; SDS= Self Rating Depression Scale; HAM-D= Hamilton Depression Rating Scale; SAS= Self-rating Anxiety scale; HADS=* [*Hospital Anxiety and Depression Scale*](https://www.svri.org/sites/default/files/attachments/2016-01-13/HADS.pdf)*; VAS= Visual Analogue Scale; ESAS= Edmond Symptoms Assessment Scale; PANAS= McGill and positive and negative affect schedule; MMSE= Mini Mental State Examination; CPT= Conners' Continuous Performance Test; PAOFI= Patient Assessment of Own Functioning Inventory*
